# Supplementary material for: The p97-UBXD8 complex regulates ER-Mitochondria contact sites by altering membrane lipid saturation and composition
Source: Nat Commun. 2023 Feb 6;14:638. doi: 10.1038/s41467-023-36298-2 (PMC9902492; doi:10.1038/s41467-023-36298-2)
Supplement: Supplementary file 1 — Supplementary Information [file 41467_2023_36298_MOESM1_ESM.pdf]

Supplementary Figure 1

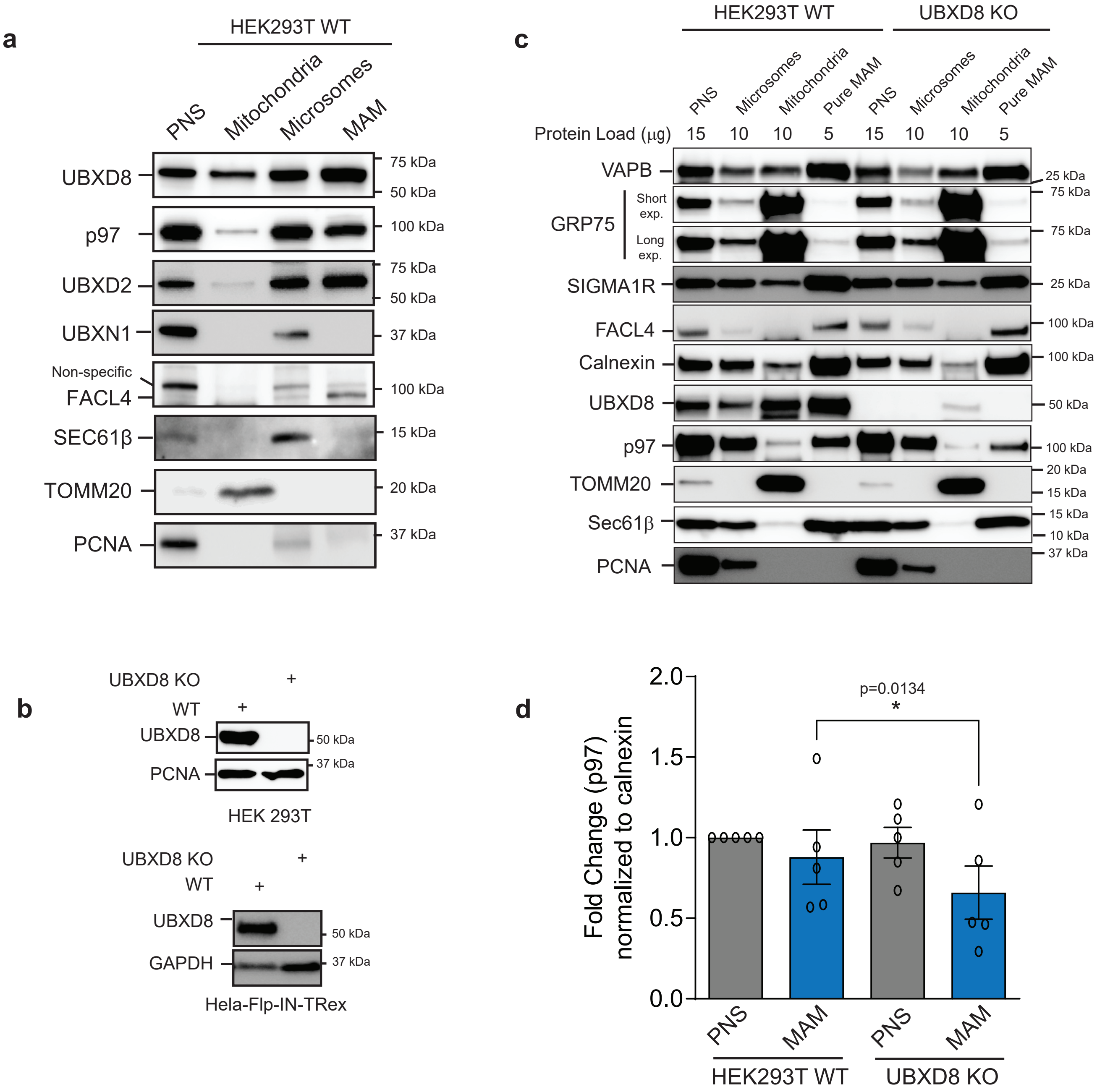

Supplementary Fig. 1. UBXD8 is enriched at ERMCS and recruits p97 to ERMCS.

A, Immunoblot of indicated proteins from subcellular fractionation of HEK-293T WT cells, PNS: post-nuclear supernatant, MAM: Mitochondria associated membrane (*n* > 3 biologically independent samples). **b**, Immunoblot of UBXD8 in CRISPR-Cas9 edited HEK-293T and HeLa-Flp-IN-T-Rex cells, KO: knockout. The experiment was independently repeated 3 times with similar result. **c**, Immunoblot of indicated proteins from subcellular fractionation of HEK-293T WT and UBXD8 KO cells (*n* > 3 biologically independent samples). **d**, Quantification of p97 band intensities, PNS *versus* MAM fractions normalized to calnexin. (n = 5 biologically independent samples). Data are means ± SEM *, $P < 0.05$. Significance was analyzed by two-tailed paired *t* test between WT *vs* UBXD8 KO MAM fractions. Source data are provided as a Source Data file.

Supplementary Figure 2

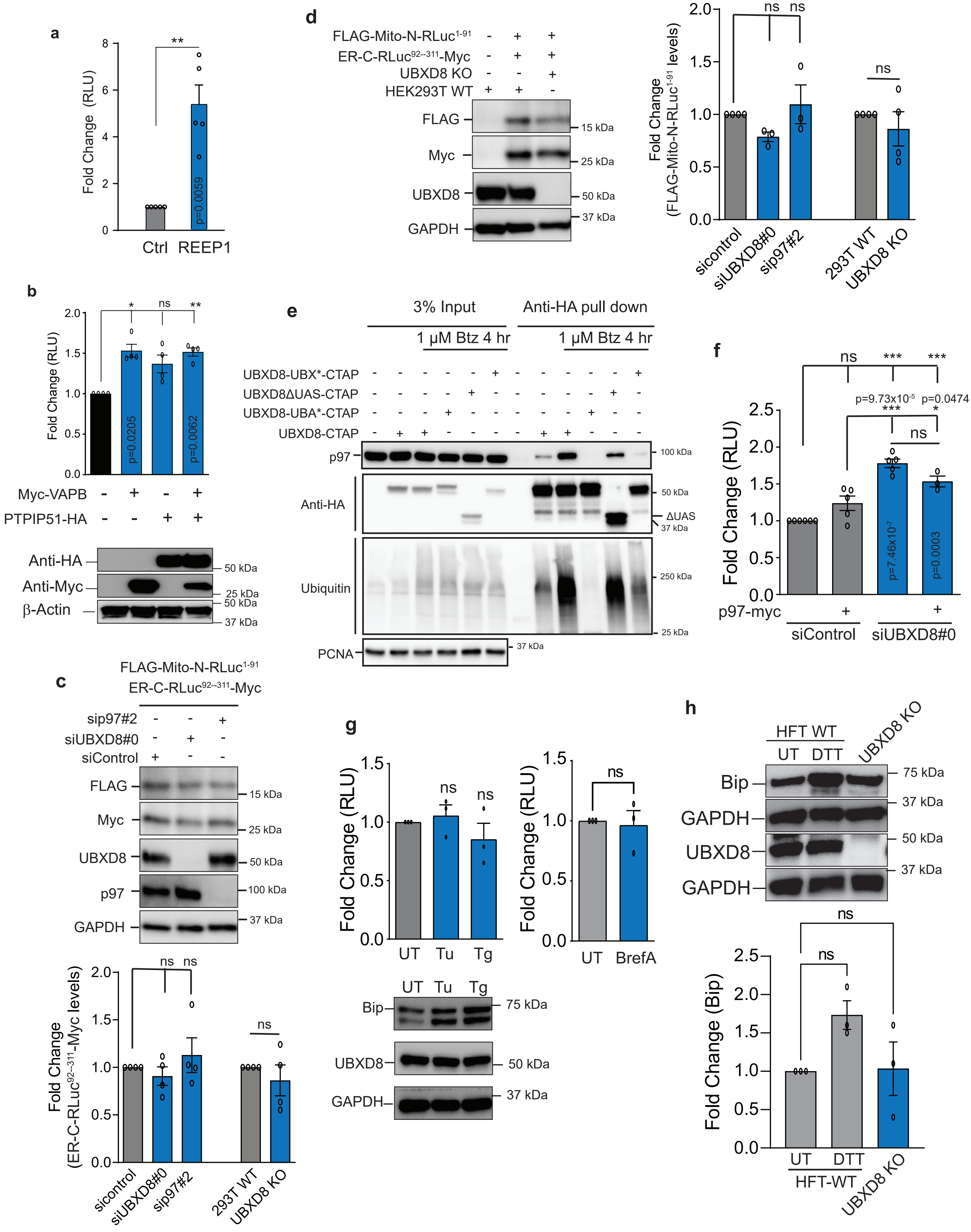

Supplementary Fig. 2. Validation of split-luciferase system, and ER stress does not alter ERMCS

a, Split luciferase assay to measure contacts in HEK-293T cells transfected with REEP1 (*n* = 5 biologically independent samples). **b**, Top: Split luciferase assay to measure contacts in HEK-293T cells transfected with indicated cDNA constructs. (*n* = 4 biologically independent samples). Bottom: Immunoblot of HEK 293T cells transfected with indicated cDNA constructs. **c,** Top panel, Immunoblot of HEK 293T cells transfected with indicated siRNAs and split-luciferase constructs. Bottom panel, quantification of band intensities of split luciferase reporters. (n = 4 biologically independent samples). **d,** Left panel, Immunoblot of HEK293T WT and UBXD8 KO cells were transfected with split-luciferase constructs. Right panel, quantification of band intensities of split luciferase reporters (n = 3 biologically independent samples except for siControl (n = 4); n = 5 biologically independent samples of WT and UBXD8 KO). **e**, Affinity purification of indicated UBXD8-HA/FLAG constructs transiently expressed in HEK-293T cells. Immunoblots of whole cell lysates and affinity purifications probed with anti-HA, anti-p97, anti-PCNA, and ubiquitin antibodies (*n* = 3 biologically independent samples). **f**, Split luciferase assay to measure contacts in HEK-293T cells transfected with indicated siRNAs and/or cDNA constructs. (n = 6, 5, 5, and 3 biologically independent samples from left to right, respectively). **g**, Top panel: Split luciferase assay to measure contacts in HEK-293T cells treated with 2.5 μM Tunicamycin (Tu), 1.5 μM Thapsigargin (Tg), for 2 hours or 10 μM Brefeldin A for 2 hours (*n* = 3 biologically independent samples). Bottom panel: Immunoblot of HEK-293T cells treated with Tu and Tg and probed for the indicated proteins. **h**, Top panel: Immunoblot of HEK-293T wildtype and UBXD8 KO cells treated with 1.5 mM Dithiothreitol (DTT) for 2 hours and probed for the indicated proteins, Bottom panel: Quantification of band intensities of Bip. UT: Untreated. (*n* = 3 biologically independent samples). Data are means ± SEM. *, **, ****P* < 0.05, 0.01, 0.0001 respectively. ns: not significant. Two-tailed paired *t*-test (**a, c** (WT vs UBXD8 KO)**,** and **d** (WT vs UBXD8 KO)); Repeated measures one-way ANOVA with Tukey's multiple comparison test (**b**); One-way ANOVA with Tukey's multiple comparison test (**c** (for siRNA treatments)**, d** (for siRNA treatments), **f-h**). Source data are provided as a Source Data file.

Supplementary Figure 3

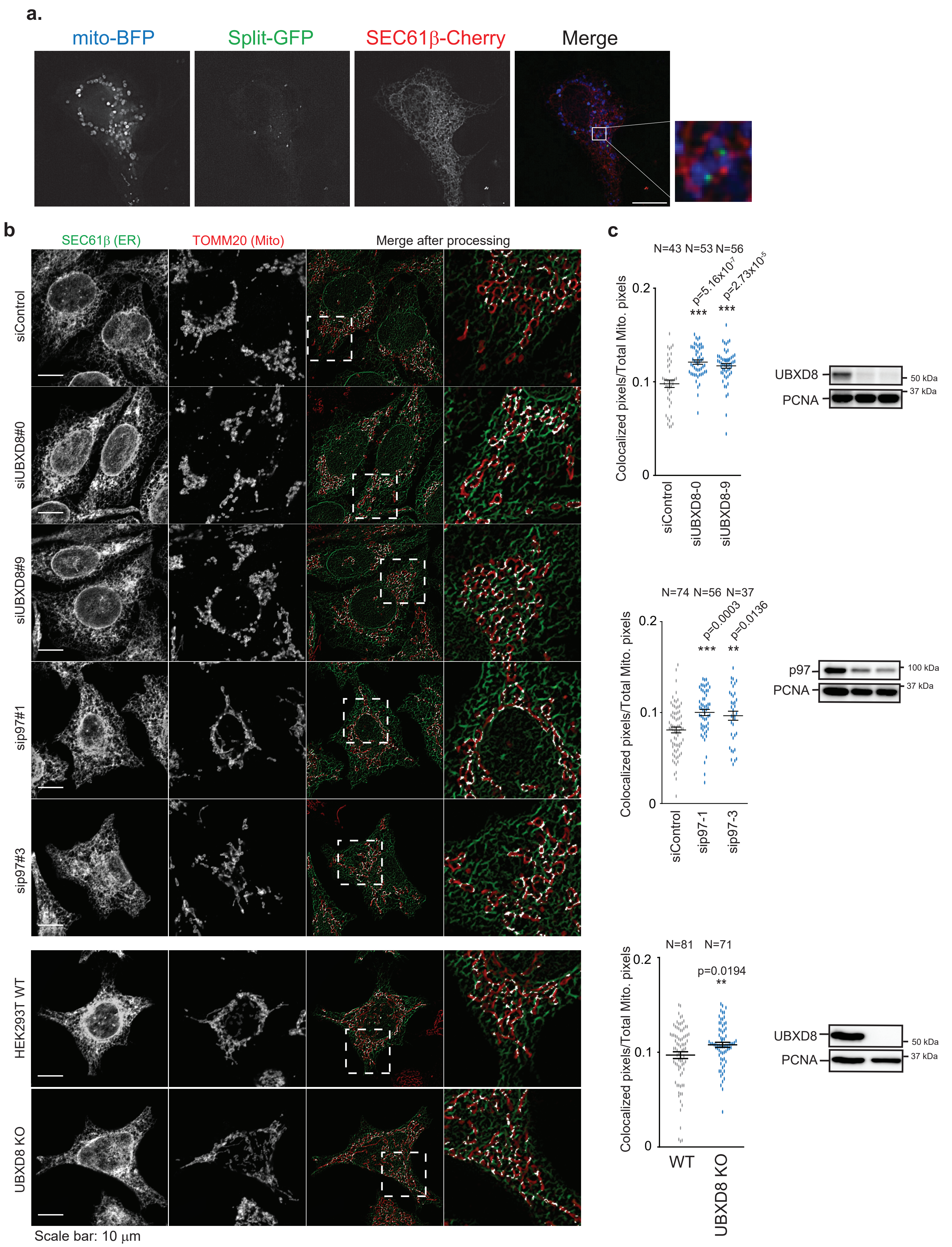

Supplementary Fig. 3. Loss of p97 and UBXD8 results in increased ERMCS

a, Representative confocal image of HeLa-Flp-IN-TRex cells transfected with mito-BFP (blue, mitochondria), Sec61β-mCherry (red, ER), and SPLICS constructs (Green, split-GFP) (Scalebar: 10μm). (n = 1 biological experiment to validate the previously reported split-GFP system[27]). **b**, Representative confocal image of HeLa-Flp-IN-TRex cells stably expressing Sec61β-eGFP (green, ER) and stained for endogenous TOMM20, (red, mitochondria). HFT cells were transfected with indicated siRNAs. Merged panels represent images after processing using an ImageJ-based analysis pipeline for the quantification of contacts between ER and mitochondria[65] (White dots are colocalized pixels; Scalebar: 10 μm). **c**, Left panels: Quantification of ER-mitochondria contacts in cells transfected with indicated siRNAs (top and middle panel) or in UBXD8 KO HeLa-Flp-IN-TRex cells (bottom panel) using assay in (**b**). Right panels: Immunoblots depicting the knockdown efficiency. (n = 3 biologically independent samples) N: numbers of cells analyzed in each condition. (Quartiles represent the upper 75th percentile and the lower 25th percentile. The line inside the box represents the median. Whiskers indicate distribution of data from minimum to maximum in a condition.). Data are means ± SEM. *, **, ***$P < 0.05$, 0.01, 0.0001 respectively. One-way ANOVA with Tukey's multiple comparison test (**c** : top and middle panel), two-tailed unpaired t test with Welch's correction (**c** : bottom panel). Source data are provided as a Source Data file.

Supplementary Figure 4

a

		Post nuclear supernatant	Mitochondria-associated membrane
	No. of proteins quantified	7556	4499
	No. of peptides quantified	56738	35371
Log_2FC=-0.65	No. of upregulated proteins in UBXD8 KO	123	108
Log_2FC=+0.65	No. of upregulated proteins in WT	226	112

b

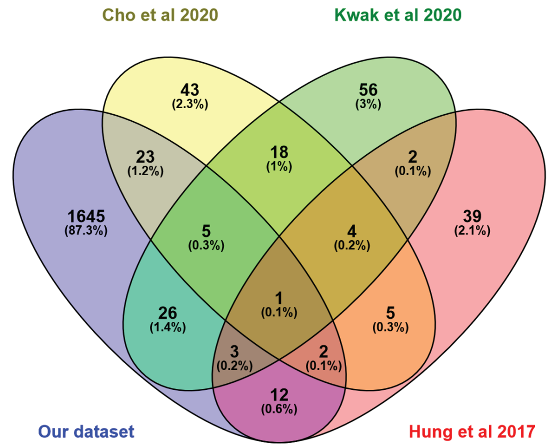

c

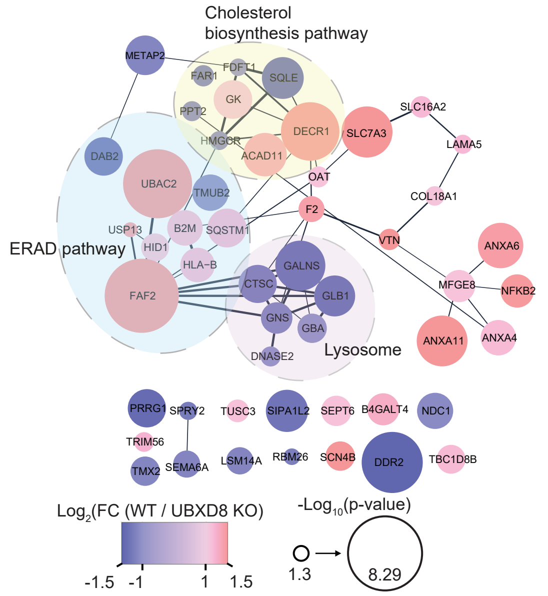

d

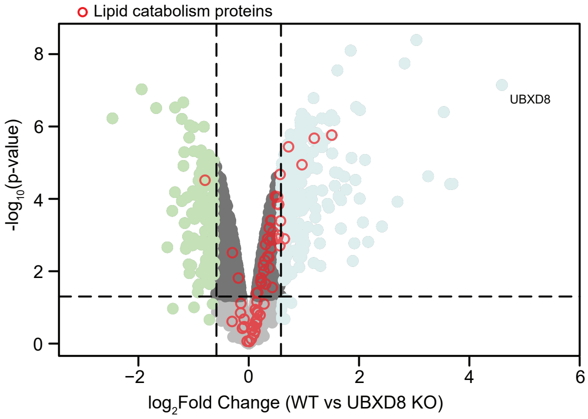

e

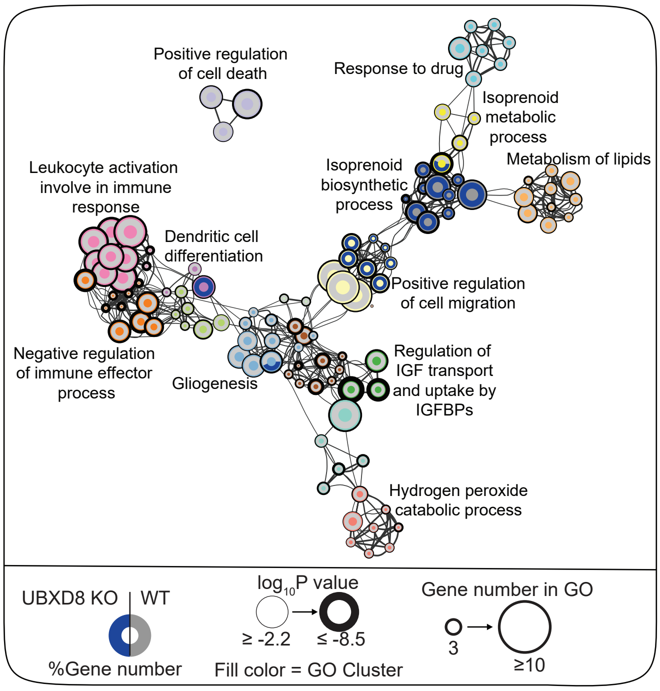

f

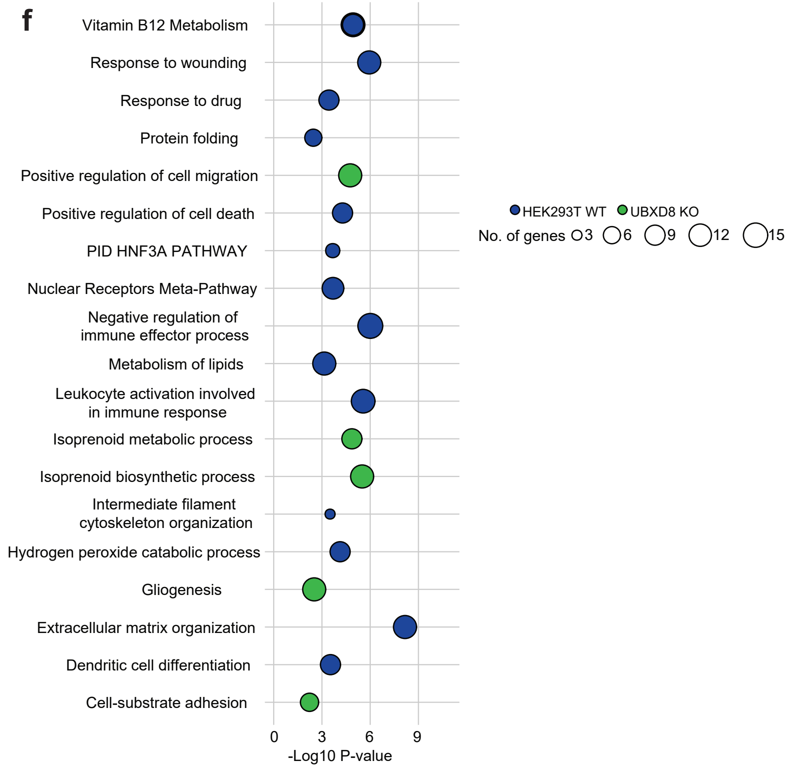

g

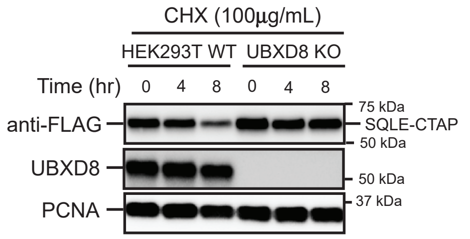

h

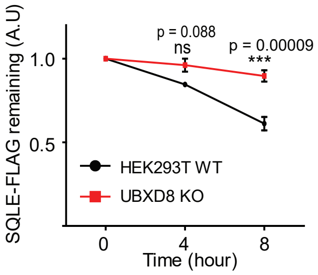

Supplementary Fig. 4. Quantitative proteomics of wildtype and UBXD8 KO contact proteome.

a, Table depicting number of proteins and peptides quantified in post-nuclear supernatant and mitochondria associated membrane fractions identified by proteomics in wildtype and UBXD8 KO cells. Number of proteins up- or downregulated at $\log_2$- fold change (FC) (wildtype/ UBXD8 KO) ± 0.65 and ±1 is indicated. **b**, Venn diagram depicting overlap of our dataset with other putative mitochondria associated membrane proteins identified by proteomics[21,30-32] **c**, Protein-protein interaction network of differentially expressed proteins from MAM fractions of HEK-293T cells involved in ERAD, cholesterol biosynthesis and lysosome function shown as clustered functional categories. Protein associations were determined using STRING database with score ≥ 0.4. Each node represents a protein belonging to enriched GO clusters as scored by Metascape. Size of node represents $-\log_{10}$-transformed *P* value and color of node represents $\log_2$- fold change (FC) (WT / UBXD8 KO). **d**, Volcano plot of the $-\log_{10}$-transformed *P* value *versus* the $\log_2$-transformed ratio of wildtype/ UBXD8 KO proteins identified in the post-nuclear supernatant of HEK-293T cells. $n = 3$ (each genotype) biologically independent samples. *P* values (**c** & **d**) were computed by empirical Bayesian statistical methods (two-tailed *t* test adjusted for multiple comparisons using Benjamini-Hochberg's correction method) available in *Limma* R package; for parameters, individual *P* values and *q* values, see Supplementary Dataset 1. **e**, Network of differentially enriched functional ontology terms shown as clustered functional ontology categories. Each node represents a functional ontology term enriched in the TMT data (**d**) as scored by Metascape and networks generated using Cytoscape v3.8.2. Size of node represents number of genes identified in each term by gene ontology (GO). Grey and Blue donuts represent percent of genes identified in each GO term in wildtype or UBXD8 KO respectively. Node outline thickness represents $-\log_{10}$-transformed *P* value of each term. The inner circle color of each node indicates the corresponding functional GO cluster. **f,** Bubble plot representing significantly enriched GO clusters identified from TMT proteomics of post-nuclear fractions in wildtype (blue) or UBXD8 KO (green) cells (**d-e**). Size of the circle indicates the number of genes identified in each cluster. **g**,**h**, Squalene epoxidase (SQLE) half-life measurements in wildtype and UBXD8 KO HEK 293T cells. FLAG-SQLE was transiently expressed, and cells were treated with 100 μg/mL cycloheximide for the indicated times. Samples were resolved on SDS-PAGE for immunoblots (**g**) and levels of SQLE were quantified and normalized to loading control PCNA (**h**); ($n = 3$ biologically independent samples). Data are means ± SEM ***: $P < 0.0001$, ns: not significant. One-way ANOVA with Tukey's multiple comparison test (**h**). Default settings on Metascape were used to perform accumulative hypergeometric statistical test to calculate the p-values (**e** & **f**). Source data are provided as a Source Data file.

Supplementary Figure 5

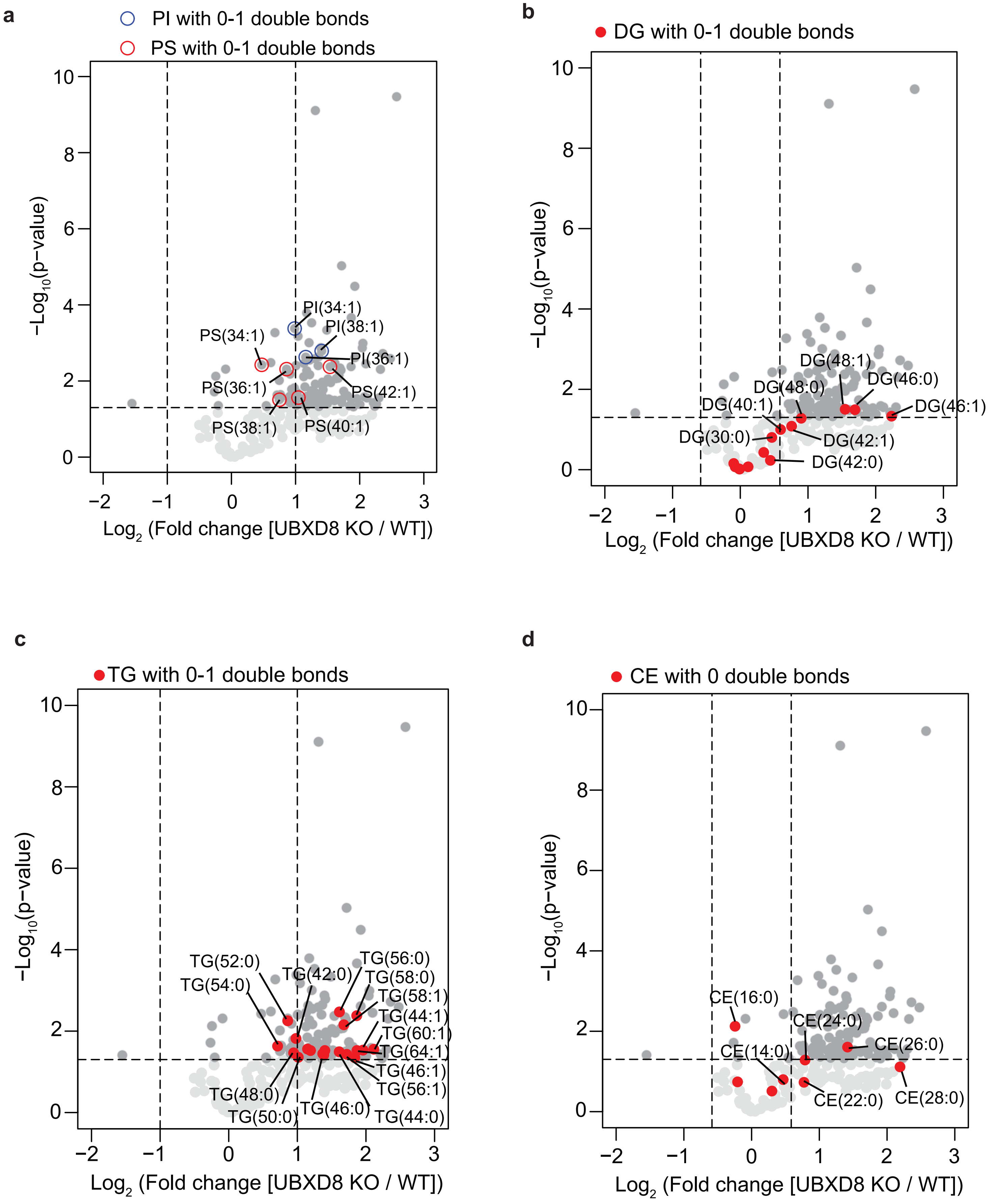

Supplementary Fig. 5. Loss of UBXD8 alters cellular lipidome with increased abundance of saturated phospholipids and neutral lipids.

a-d, Volcano plot of the total phospholipid and neutral lipid species identified using lipidomics of whole cell extracts of HEK-293T cells ($-\log_{10}$-transformed P value versus the $\log_2$-transformed ratio of UBXD8 KO : wildtype). The same plot is shown in each panel with select saturated or monounsaturated lipids are shown for PS (red outline) and PI (blue outline) species (**a**), DG species (red filled circles) (**b**), TG species (red filled circles) (**c**), and CE (red filled circles) (**d**). Lipids were measured by LC-MS/MS following normalization by total protein amount. ($n \geq 3$ biologically independent experiments were performed, each with duplicate samples). Statistical analysis (in **a-d**) was performed on the $\log_2$ transformed relative fold change values (UBXD8 KO relative to WT) using independent two-tailed *t* tests and Benjamini-Hochberg correction in R stats package (p-values are listed in Supplemental Dataset 2). PI, phosphatidylinositol; PS, phosphatidylserine; DG, Diacylglycerol; TG, Triacylglycerol; and CE, Cholesteryl esters.

Supplementary Figure 6

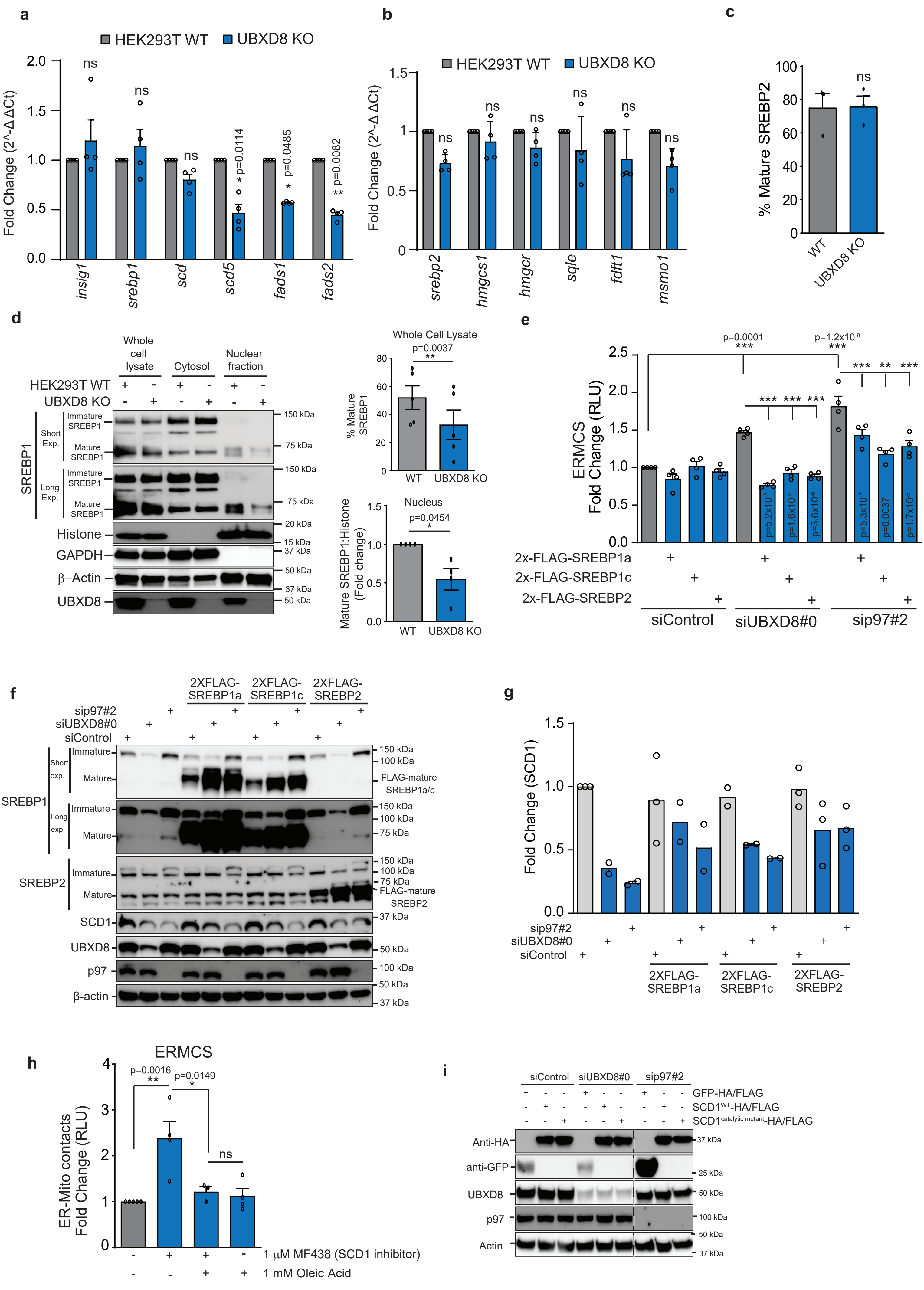

Supplementary Fig. 6. Diminished SREBP pathway activation in UBXD8 KO cells

a-b, Real-time quantitative PCR of SREBP1 target genes including lipid desaturases (**a**), and SREBP2 target genes (**b**) (n = 4 biologically independent samples). **c,** Band intensity quantifications of mature SREBP2 in wildtype and UBXD8 KO HEK-293T cells corresponding to **Fig 6a** (n = 3 biologically independent samples). **d**, Left panel: Immunoblot of whole cell, nuclear, and cytosolic fractions of HEK293T WT and UBXD8 KO cells with indicated proteins. Right panel: Band intensity quantifications of mature SREBP1 in whole cell and nuclear extracts. (n = 5 (Whole cell lysate panel); n = 4 (Nucleus panel) biologically independent samples). **e,** Split luciferase assay to measure contacts in HEK-293T cells transfected with siRNAs to UBXD8 or p97 and indicated 2X-FLAG-tagged mature SREBP1a, 1c, and 2 constructs. RLU: relative luminescence unit. (n = 4 biologically independent samples). **f**, Immunoblot of indicated proteins in HEK-293T cells transfected with siRNAs to UBXD8 or p97 and indicated 2X-FLAG-tagged mature SREBP1a, 1c, and 2 constructs. Immunoblots were probed with antibodies to SREBP1 and 2 to visualize immature and transfected mature forms. **g**, Quantification of band intensities of SCD1 desaturase corresponding to immunoblot in **f**. (n = 3, 2, 2, 3, 2, 2, 2, 2, 2, 3, 3, and 3 biologically independent samples from left to right, respectively). **h**, Split luciferase assay to measure contacts in HEK 293T cells treated with SCD1 inhibitor MF438 at 1 μM for 4 hours. Cells were also treated with oleic acid for 4 hours as indicated. (n = 5, 4, 3, and 4 biologically independent samples from left to right, respectively). **i,** Immunoblot of indicated proteins in HEK293T cells transfected with indicated siRNAs and wildtype or catalytically dead mutant of SCD1. GFP-HA/FLAG was transfected as a negative control. Related for Fig. **6i**. (n = 3 independent biological replicates). Data are means ± SEM. *, **, *** $P < 0.05, 0.01, 0.0001$ respectively. ns: not significant. One-way ANOVA with Dunnett's multiple comparison test (**a, b**), Two-tailed paired t test with Welch's correction (**c & d**) or One-way ANOVA with Tukey's multiple comparison test (**e** and **h**). Source data are provided as a Source Data file.

Supplementary Figure 7

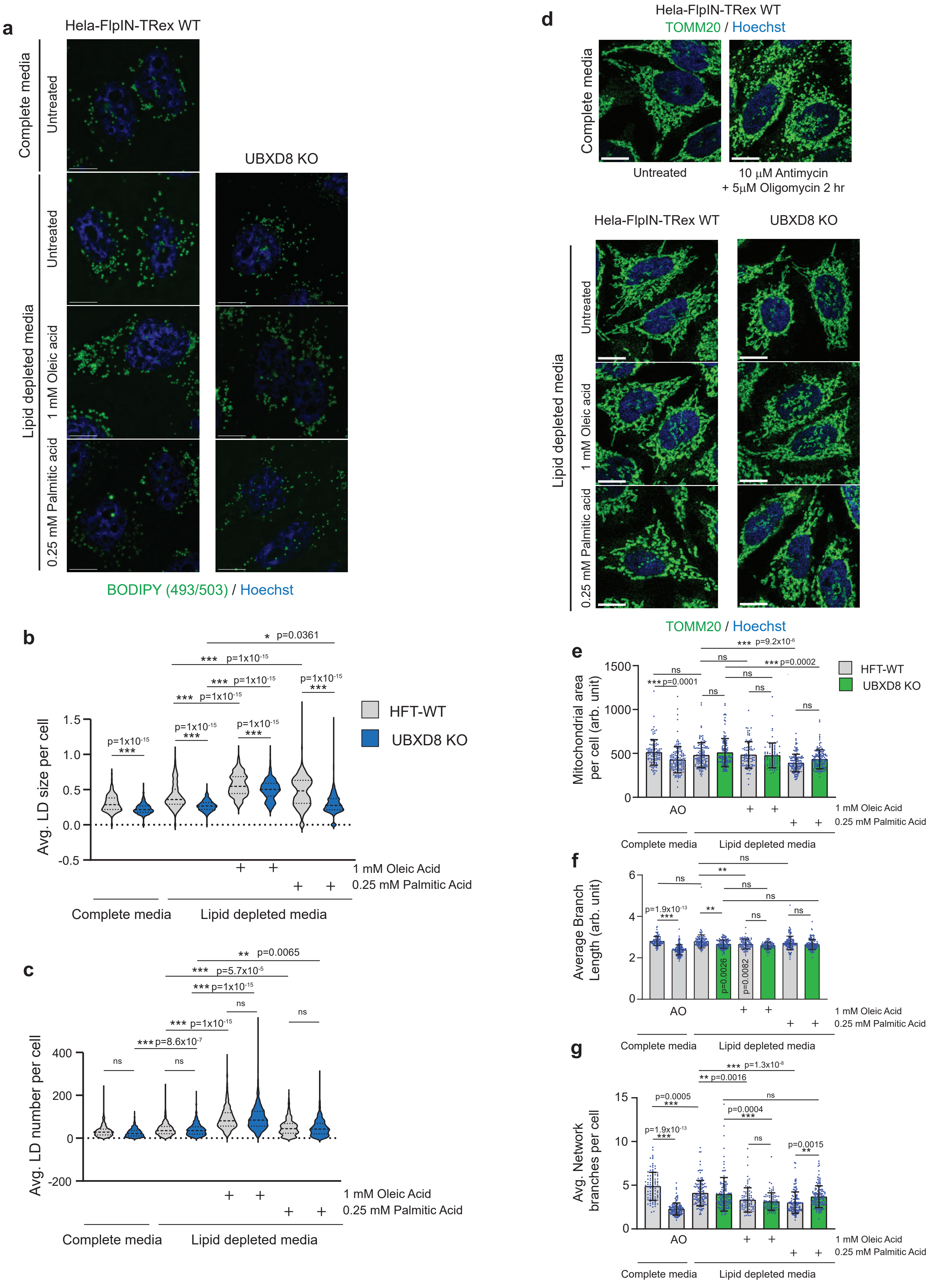

Supplementary Fig. 7. Lipid droplets and mitochondrial morphology are comparable between WT and UBXD8 KO.

a, Representative fluorescence microscopy images of HFT wildtype and UBXD8 KO stained for lipid droplets using BODIPY 493/503 dye. Scalebar: 10 μm. Cells were lipid depleted and/or supplemented with indicated concentrations of oleic acid and palmitic acid for 4 hours. **b-c**, Quantification of lipid droplets in (**a**) using Aggrecount[66,67] to represent lipid droplet size per cell (**b**) and lipid droplets number per cell (**c**). (n = 3 biologically independent samples). **d,** Representative immunofluorescence microscopy images of HFT wildtype and UBXD8 KO stained for mitochondria using anti-TOMM20 antibody. Scalebar: 20 μm. HFT wildtype cells were treated with 10 μM of Antimycin and 5 μM Oligomycin for 2 hrs. WT and UBXD8 KO cells were lipid depleted and/or supplemented with indicated concentrations of oleic acid and palmitic acid for 4 hours. (n = 3 biologically independent replicates. Quantifications and cell numbers are provided in the legends of **e-g**). **e-g**, Mitochondrial morphology measurement were performed using MiNA[68]. Bar graphs represent mitochondrial area per cell **(e)**, average branch length **(f)**, and average network branches per cell **(g)**. (Each data point in the plots **e-f** represents a cell. The cell numbers used for quantification are 101, 135, 121, 127, 87, 82, 132, and 135 from left to right, respectively across n = 3 biologically independent replicates). Data are means ± SEM. *, **, ****P* < 0.05, 0.01, 0.0001 respectively, ns: not significant. One-way ANOVA with Tukey’s multiple comparison test. Source data are provided as a Source Data file.

Supplementary Figure 8

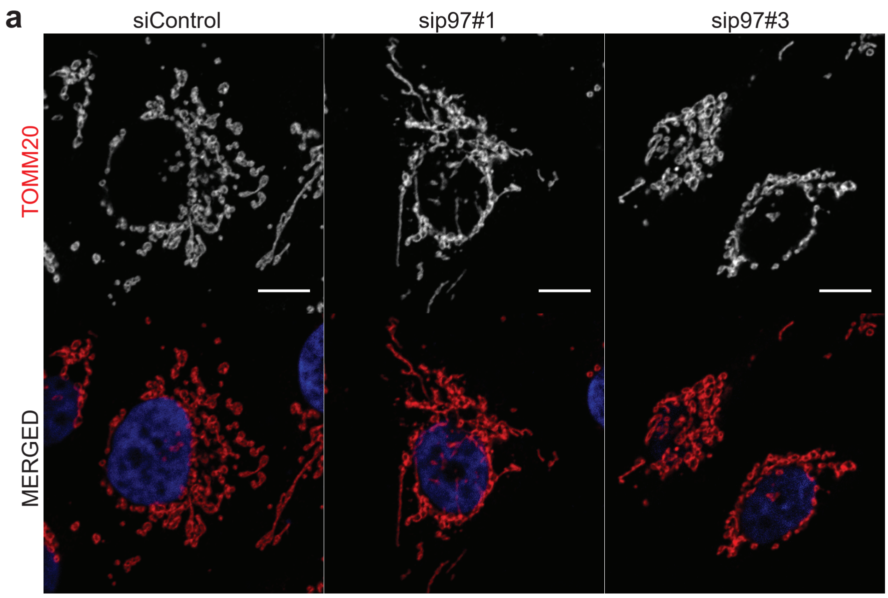

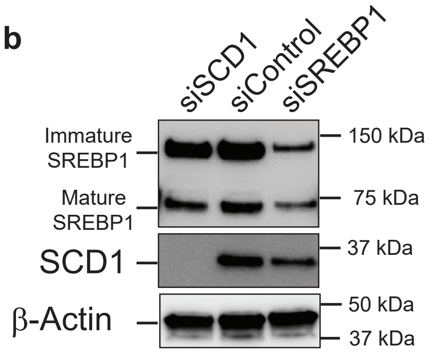

Supplementary Fig. 8. p97 depletion does not affect mitochondrial morphology.

a, Representative immunofluorescence microscopy images of HFT wildtype and p97 depleted cells stained for mitochondria using anti-TOMM20 antibody. Scalebar: 10 μm. (n = 3 biologically independent samples) **b,** Representative immunoblot of indicated proteins in HEK-293T cells transfected with siRNAs to SREBP1 and SCD1. (n = 2 biologically independent samples). Source data are provided as a Source Data file.

Supplementary Table 1: List of Constructs used in this study

Construct	Vector backbone	Tag	Insert	Insert
pCDNA-FRT/TO-N-eGFP-UBXD8	pCDNA-FRT/TO-NeGFP-GaW	N-eGFP	UBXD8	This study
pHAGE-UBXD8-CTAP	pHAGE-C-HA/FLAG	C-HA/FLAG	UBXD8	Raman., et al. Mol Cell 44, 72-84 (2011).
pHAGE-FAF2-CTAP_siRescue	pHAGE-C-HA/FLAG	C-HA/FLAG	UBXD8	This study
pHAGE-FAF2_delUAS-CTAP_siRescue	pHAGE-C-HA/FLAG	C-HA/FLAG	UBXD8	This study
pHAGE-FAF2_UBX*-CTAP_siRescue	pHAGE-C-HA/FLAG	C-HA/FLAG	UBXD8	This study
pHAGE-FAF2_UBA*-CTAP_siRescue	pHAGE-C-HA/FLAG	C-HA/FLAG	UBXD8	This study
pHAGE-Sec61-C-eGFP	pHAGE-C-HA/FLAG	C-eGFP	SEC61β	This study
pHAGE-Insig1-CTAP	pHAGE-C-HA/FLAG	C-HA/FLAG	INSIG1	This study
pcDNA3-Mit-NRluc91	pcDNA3.1 TOPO		*Renilla* luciferase 8 (*RLuc8*) *1-91 a.a*	Kind gift from Jeffrey A. Golden (Cho.,et al. J Biol Chem 292, 16382-16392 (2017))
pcDNA3-CRluc92-ER	pcDNA3.1 TOPO		*Renilla* luciferase 8 (*RLuc8*) *92-311 a.a*	Kind gift from Jeffrey A. Golden (Cho.,et al. J Biol Chem 292, 16382-16392 (2017))
pCAG-IRES-GFP	pCAG-IRES-GFP	GFP		Kind gift from Jeffrey A. Golden (Cho.,et al. J Biol Chem 292, 16382-16392 (2017))
pCAG-IRES-REEP1-GFP	pCAG-IRES-GFP	GFP	REEP1	Kind gift from Jeffrey A. Golden (Cho.,et al. J Biol Chem 292, 16382-16392 (2017))
2X-FLAG-SREBP1a	pcDNA3.1+	2X-FLAG	Mature SREBP1a	Kind gift from Timothy Osborne (Addgene plasmid # 26801)
2X-FLAG-SREBP1c	pcDNA3.1+	2X-FLAG	Mature SREBP1c	Kind gift from Timothy Osborne (Addgene plasmid # 26802)
2X-FLAG-SREBP2	pcDNA3.1+	2X-FLAG	Mature SREBP2	Kind gift from Timothy Osborne (Addgene plasmid # 26807)
pHAGE-SCD1-CTAP	pHAGE-C-HA/FLAG	C-HA/FLAG	SCD1	This study

pHAGE-SCD1-mut-CTAP	pHAGE-C-HA/FLAG	C-HA/FLAG	SCD1	This study
pHAGE-SQLE-CTAP	pHAGE-C-HA/FLAG	C-HA/FLAG	SQLE	This study
pDEST MYC p97 si RESCUE	pDEST N Myc	N Myc	p97	Raman., et al. Mol Cell 44, 72-84 (2011).
pDEST MYC p97 K254A siRESCUE	pDEST N Myc	N Myc	p97	Raman., et al. Mol Cell 44, 72-84 (2011).
pDEST MYC p97 E305Q siRESCUE	pDEST N Myc	N Myc	p97	Raman., et al. Mol Cell 44, 72-84 (2011).
pDEST MYC p97 K524A siRESCUE	pDEST N Myc	N Myc	p97	Raman., et al. Mol Cell 44, 72-84 (2011).
pDEST MYC p97 E578Q siRESCUE	pDEST N Myc	N Myc	p97	Raman., et al. Mol Cell 44, 72-84 (2011).
mito-BFP	pAcGFP1-N1	BFP (C terminal on backbone)	COX4, aa1-21	Kind gift from Gia Voeltz (Addgene plasmid # 49151)
GFP-MAPPER	pEGFP-C1	GFP (N terminal on insert)	ER signal peptide	Kind gift from Jen Liou (Addgene plasmid # 117721)
ER-short-GFP$_{\beta 11}$	pCDNA3.1(+)		split-GFP	Kind gift from Tito Cali and Marisa Brini (Cieri, D., et al. Cell Death Differ 25, 1131–1145 (2018)).
OMM-GFP$_{1-10}$	pCDNA3.1(+)		split-GFP	Kind gift from Tito Cali and Marisa Brini (Cieri, D., et al. Cell Death Differ 25, 1131–1145 (2018)).
pCIneo-PTPIP51-HA	pCI-Neo	HA	PTPIP51	Kind gift from Christopher Miller (Stoica, R. et al. Nat Commun 5, 3996 (2014)).
pCIneo-VAPB-Myc	pCI-Neo	Myc	VAPB	Kind gift from Christopher Miller (Stoica, R. et al. Nat Commun 5, 3996 (2014)).
pHAGE-Sec61-mCherry	pHAGE-mCherry	mCherry	SEC61β	This study
pHAGE-UBXD8-mCherry	pHAGE-mCherry	mCherry	UBXD8	This study
