# Supplementary material for: The p97-UBXD8 complex regulates ER-Mitochondria contact sites by altering membrane lipid saturation and composition
Source: Nat Commun. 2023 Feb 6;14:638. doi: 10.1038/s41467-023-36298-2 (PMC9902492; doi:10.1038/s41467-023-36298-2)
Supplement: Supplementary file 3 — Description of Additional Supplementary Files [file 41467_2023_36298_MOESM3_ESM.pdf]

File name: Supplementary Dataset 1

Description: Quantitative TMT proteomic data of post-nuclear supernatants and MAM fractions.

File name: Supplementary Dataset 2

Description: Lipidomic data of whole cell lysates and MAM fractions.
