# Supplementary material for: The p97-UBXD8 complex regulates ER-Mitochondria contact sites by altering membrane lipid saturation and composition
Source: Nat Commun. 2023 Feb 6;14:638. doi: 10.1038/s41467-023-36298-2 (PMC9902492; doi:10.1038/s41467-023-36298-2)
Supplement: Supplementary file 6 — Reporting Summary [file 41467_2023_36298_MOESM6_ESM.pdf]

Corresponding author(s): Malavika Raman

Last updated by author(s): Jan 2, 2023

# Reporting Summary

Nature Portfolio wishes to improve the reproducibility of the work that we publish. This form provides structure for consistency and transparency in reporting. For further information on Nature Portfolio policies, see our Editorial Policies and the Editorial Policy Checklist.

## Statistics

For all statistical analyses, confirm that the following items are present in the figure legend, table legend, main text, or Methods section.

| n/a | Confirmed | |
|---|---|---|
| ☐ | ☒ | The exact sample size (*n*) for each experimental group/condition, given as a discrete number and unit of measurement |
| ☐ | ☒ | A statement on whether measurements were taken from distinct samples or whether the same sample was measured repeatedly |
| ☐ | ☒ | The statistical test(s) used AND whether they are one- or two-sided<br>*Only common tests should be described solely by name; describe more complex techniques in the Methods section.* |
| ☒ | ☐ | A description of all covariates tested |
| ☐ | ☒ | A description of any assumptions or corrections, such as tests of normality and adjustment for multiple comparisons |
| ☐ | ☒ | A full description of the statistical parameters including central tendency (e.g. means) or other basic estimates (e.g. regression coefficient) AND variation (e.g. standard deviation) or associated estimates of uncertainty (e.g. confidence intervals) |
| ☐ | ☒ | For null hypothesis testing, the test statistic (e.g. *F*, *t*, *r*) with confidence intervals, effect sizes, degrees of freedom and *P* value noted<br>*Give P values as exact values whenever suitable.* |
| ☐ | ☒ | For Bayesian analysis, information on the choice of priors and Markov chain Monte Carlo settings |
| ☒ | ☐ | For hierarchical and complex designs, identification of the appropriate level for tests and full reporting of outcomes |
| ☒ | ☐ | Estimates of effect sizes (e.g. Cohen's *d*, Pearson's *r*), indicating how they were calculated |

*Our web collection on statistics for biologists contains articles on many of the points above.*

## Software and code

Policy information about availability of computer code

| | |
|---|---|
| Data collection | 1. AggreCount, an automated image analysis tool written in the ImageJ macro language based on the FIJI distribution of ImageJ (version 1.52p or later) was used for lipid droplet analyses (https://aggrecount.github.io/).<br>2. MiNA v3.0.1 (Mitochondrial Network Analysis) is a ImageJ based utility to aid in quantitatively describing the appearance of mitochondrial morphology (https://github.com/StuartLab/MiNA).<br>3. The code for ImageJ macro used for quantifying ER-mitochondrial contact sites has been made available on GitHub (https://github.com/theramanlab/ganji2022ubxd8).<br>4. Bands on PVDF membranes were detected using Clarity Western ECL detection kit (Biorad) and captured on BioRad Chemidoc MP imaging system or SYNGENE.<br>5. Immunofluorescence images were acquired on Nikon Eclipse 80i fluorescence microscope, Nikon Eclipse Ti2 widefield inverted microscope, and Nikon A1R microscope.<br>6. Electron Microscopy images were acquired on FEI Tecnai spirit at 80KV and photographed with an AMT CCD camera. |
| Data analysis | 1. Graph Pad Prism version 5.01, 9.2.0, 9.3.1, & 9.4.1 for Windows, Graph Pad Software, San Diego California USA (www.graphpad.com) was used for data analysis.<br>2. Cytoscape software (v3.8.2) ; RStudio software (v1.4.1103), including packages R statistical package (v4.0.3), Bioconductor (v3.12; BiocManager 1.30.10), hrbrthemes (v0.8.0), viridis (v0.6.1), dplyr (v.1.0.7), and ggplot2 (v 3.3.5) were used for data analyses and visualizations wherever applicable.<br>3. For immunofluorescence based quantification studies in Supplementary Figure 1d & 1e ImageJ-based macro was written using a previously published protocol. This macro was used to keep the processing of images uniform across all the samples. The code for macro has been uploaded on GitHub.<br>4. For immunofluorescence based quantification studies in Supplementary Figure 7a, 7b & 7c a previously published ImageJ-based macro, called as Aggrecount was used.<br>5. For immunofluorescence based quantification studies in Supplementary Figure 7d, 7e, 7f, & 7g a previously published ImageJ-based macro, called as MiNA was used. Both the macros were used to keep the processing of images uniform across all the samples. |

6. Where indicated, the contrast of the whole Western blot image was adjusted using Adobe Photoshop v23.5 Release, Bands on Western blots were quantified using ImageJ (version 1.52p or later).

For manuscripts utilizing custom algorithms or software that are central to the research but not yet described in published literature, software must be made available to editors and reviewers. We strongly encourage code deposition in a community repository (e.g. GitHub). See the Nature Portfolio guidelines for submitting code & software for further information.

## Data

Policy information about availability of data

All manuscripts must include a data availability statement. This statement should provide the following information, where applicable:

- Accession codes, unique identifiers, or web links for publicly available datasets
- A description of any restrictions on data availability
- For clinical datasets or third party data, please ensure that the statement adheres to our policy

The accession codes and unique identifiers are provided in the manuscript. The mass spectrometry proteomics data related to Figure 4 and Supplementary Figure 4 have been deposited to the ProteomeXchange Consortium via the PRIDE partner repository with the dataset identifier PXD039061. All raw lipidomic data is available in supplementary Dataset 2. The mass spectrometry lipidomics data is available at the NIH Common Fund's National Metabolomics Data Repository (NMDR) website, the Metabolomics Workbench, (https://www.metabolomicsworkbench.org), where it has been assigned Project ID PR001559 with StudyIDs ST002421 (for whole cell lipidomics) and ST002422 (MAM fraction lipidomics). The data can be accessed directly via it's Project DOI: 10.21228/M85X3W. This work is supported by NIH grant, U2C- DK119886. Any other data is available from the corresponding author upon request. Source data are provided with this paper.

# Field-specific reporting

Please select the one below that is the best fit for your research. If you are not sure, read the appropriate sections before making your selection.

☒ Life sciences ☐ Behavioural & social sciences ☐ Ecological, evolutionary & environmental sciences

For a reference copy of the document with all sections, see nature.com/documents/nr-reporting-summary-flat.pdf

# Life sciences study design

All studies must disclose on these points even when the disclosure is negative.

| | |
|---|---|
| Sample size | No statistical method was used to predetermine sample size. In general, all relevant studies were repeated atleast 3 independent times. Detailed n number are provided in the figures and/or figure legends. Representative image from these repeats were presented, where applicable in the figures. For mice studies the sample size were chosen based on previous studies by Weihl group (PMID: 34289347; PMID: 30100055). For proteomics studies, sample size were chosen based on previously published studies by the Raman, and Gygi groups (PMID: 26389662; PMID: 28375945). For lipidomics sample size were chosen based on previously published studies by the Purdy group ( PMID: 31391267; PMID: 33947752). |
| Data exclusions | No data were excluded from the analyses. |
| Replication | More than or equal to 3 biologically independent experiments, except for Supplementary figure 6g-h, which were performed 2 times, were used as replicates for reproducibility of experimental findings. The number replicates used for each data are mentioned in the figure legends. |
| Randomization | This study does not involve allocation of samples into experimental groups. So, randomization is not applicable. Imaging fields for microscopy-base image acquisition were chosen randomly. |
| Blinding | For immunoflourescene based microscopy, fields of cells were chosen based on the Hoechst (nuclei) channel to prevent biased image acquisition. Followed by quantification studies using ImageJ-based macros to keep the processing of images uniform across all the samples. The code for macro will be uploaded to GitHub wherever applicable. |

# Reporting for specific materials, systems and methods

We require information from authors about some types of materials, experimental systems and methods used in many studies. Here, indicate whether each material, system or method listed is relevant to your study. If you are not sure if a list item applies to your research, read the appropriate section before selecting a response.

## Materials & experimental systems

| n/a | Involved in the study |
| --- | --- |
| ☐ | ☒ Antibodies |
| ☐ | ☒ Eukaryotic cell lines |
| ☒ | ☐ Palaeontology and archaeology |
| ☐ | ☒ Animals and other organisms |
| ☒ | ☐ Human research participants |
| ☒ | ☐ Clinical data |
| ☒ | ☐ Dual use research of concern |

## Methods

| n/a | Involved in the study |
| --- | --- |
| ☒ | ☐ ChIP-seq |
| ☒ | ☐ Flow cytometry |
| ☒ | ☐ MRI-based neuroimaging |

# Antibodies

Antibodies used

The p97 (10736-1-AP; WB: 1:2000), UBXD8 (16251-1-AP; WB: 1:2000), FACL4 (22401-1-AP; WB: 1:2000), UBXD2 (21052-1-AP; WB: 1:2000), HRD1 (13473-1-AP; WB: 1:2000), Sec61b(51020-2-AP; WB: 1:2000), Calnexin (10427-2-AP; WB: 1:2000), UBXN1 (16135-1-AP; WB: 1:3000), SREBP1 (14088-1-AP; WB: 1:2000), SREBP2 (28212-1-AP; WB: 1:2000), FADS1 (10627-1-AP; WB: 1:2000), anti-GFP (66002-1-AP; WB: 1:2000), AMFR/GP78 (16675-1-AP; WB: 1:2000), GRP75 (14887-1-AP; WB: 1:2000), VAPB (14477-1-AP; WB: 1:2000), and SCD1 (23393-1-AP; WB: 1:2000) antibodies were from Proteintech Inc. The TIMM23 (H-8; sc514463; WB: 1:2000), TOMM20 (F-10; sc17764; WB: 1:2000), TOMM70 (A-8; sc390545; WB: 1:2000), pan-ubiquitin (P4D1; sc8017; WB: 1:2000), c-Myc (9E10; sc40; WB: 1:2000), b-Actin (AC-15; sc69879; WB: 1:2000), SIGMA1R (B-5; sc137075; WB: 1:2000), GAPDH (O411; sc47724; WB: 1:2000), and PCNA (PC10; sc56; WB: 1:2000) antibodies were obtained from Santa Cruz Biotechnologies. LC3B (D11; 3868S; WB: 1:1000), and BiP (C50B12; 3177T; WB: 1:2000) were from Cell Signaling Technologies. p97 (A300-589A; WB: 1:2000) was from Bethyl laboratories. The following antibodies Histone-H3 (ab1791; Abcam; WB: 1:2000), UBXD7 (PA5-61972; Invitrogen; WB: 1:2000), anti-HA (16B12; MMS-101P, Covance; WB: 1:2500), anti-FLAG (M2; F3165 Sigma Aldrich; WB: 1:5000), were used for immunoblotting. HRP conjugated anti-rabbit (cat# W401B; WB: 1:10,000) and anti-mouse (cat# W402B; WB: 1:10,000) secondary antibodies were from Promega. Goat anti-Mouse IgG (H+L) Cross-Adsorbed Secondary Antibody, Alexa Fluor™ 568 (Catalog # A-11004; IF: 1:10,000; PMID: 36350286), and Goat anti-Mouse IgG (H+L) Cross-Adsorbed Secondary Antibody, Alexa Fluor™ 488 (Catalog # A-11001; IF: 1:10,000) were purchased from ThermoFisher Scientific.

Validation

All abtibodies were procured from commercial sources, which were validated by the manufacturer and cited by other research groups. Provided the PMIDs for each of the antibodies used.
Rabbit p97 (Proteintech; Cat# 10736-1-AP; Specificity: Human, Mouse, and rat; for WB; PMID: 34038735)
Rabbit UBXD8 (Proteintech; Cat# 16251-1-AP; Specificity: Human, Mouse, and rat; for WB; PMID: 29514927),
Rabbit FACL4 (Proteintech; Cat# 22401-1-AP; Specificity: Human, and rat; for WB; PMID: 31591388),
Rabbit UBXD2 (Proteintech; Cat# 21052-1-AP; Specificity: Human, Mouse, and rat; for WB; PMID: 30204128),
Rabbit HRD1 (Proteintech; Cat# 13473-1-AP; Specificity: Human, Mouse, and rat; for WB; PMID: 29269412),
Rabbit Sec61beta (Proteintech; Cat# 51020-2-AP; Specificity: Human, and Mouse; for WB; PMID: 34453725),
Rabbit Calnexin (Proteintech; Cat# 10427-2-AP; Specificity: Hamster, human, monkey, mouse, pig, rat; for WB; PMID: 31006537),
Rabbit UBXN1 (Proteintech; Cat# 16135-1-AP; Specificity: Human, rat, and Mouse; for WB; PMID: 29685906),
Rabbit SREBP1 (Proteintech; Cat# 14088-1-AP; Specificity: bovine, chicken, goat, human, pig; for WB; PMID: 30928092),
Rabbit SREBP2 (Proteintech; Cat# 28212-1-AP; Specificity: Human; for WB; PMID: 32500071),
Rabbit FADS1 (Proteintech; Cat# 10627-1-AP; Specificity: Human, and Mouse; for WB; PMID: 32982427 ),
Rabbit AMFR/GP78 (Proteintech; Cat# 16675-1-AP; Specificity: Human, Mouse, and rat; for WB; PMID: 26949185),
Rabbit GRP75 (Proteintech: 14887-1-AP; Specificity: Human, Mouse, and rat; for WB; PMID: 24856930),
Rabbit VAPB (Proteintech: 14477-1-AP; Specificity: Human, Mouse, Monkey, and rat; for WB; PMID: 34873283),
Mouse anti-GFP (Proteintech; Cat# 66002-1-AP; Specificity: Recombinant protein; for WB; PMID: 33731709)
Rabbit SCD1 (Proteintech; Cat# 23393-1-AP; Specificity: Human, rat, and Mouse; for WB; PMID: 33346941).
Mouse TIMM23 (Santacruz; clone: H-8; cat# sc514463; Specificity: Human, rat, and Mouse; for WB; PMID: 33828088),
Mouse TOMM20 (Santacruz; clone: F-10; Cat# sc17764; Specificity: Human, rat, and Mouse; for WB; PMID: 33535046 ),
Mouse TOMM70 (Santacruz; clone: A-8; Cat# sc390545; Specificity: Human, rat, and Mouse; for WB; PMID: 33597756),
Mouse pan-ubiquitin (Santcruz; clone: P4D1; Cat# sc8017; Specificity: Human, rat, drosophila, and Mouse; for WB; PMID: 33990575),
Mouse c-Myc (Santacruz; clone: 9E10; Cat# sc40; Specificity: mouse, rat, human, monkey, feline and canine; for WB; PMID: 27126587),
Mouse SIGMA1R (Santacruz; clone: B-5; Cat# sc137075; Specificity: mouse, rat, and human; for WB; PMID: 35508606)
Mouse beta-Actin (Santacruz; clone: AC-15; Cat# sc69879; Specificity: broad species origin, for WB; PMID: 34608126),
Mouse GAPDH (Santacruz; clone: O411; Cat# sc47724; Specificity: Human; for WB; PMID: 34133922),
Mouse PCNA (Santacruz; clone: PC10; Cat# sc56; Specificity: mouse, rat, human, insect and S. pombe; for WB; PMID: 33497360 ),
Rabbit BiP (CST; clone: C50B12; Cat# 3177T; Specificity: Human, and Mouse; for WB; PMID: 34099666)
Rabbit p97 (Bethyl; Cat# A300-589A; Speicificity: Human, and Mouse; for WB and IP; PMID: 21981919),
Mouse HA (Covance, clone 16B12, Cat #:MMS101P, Specificity: recombinant protein; for WB; PMID: 19615732 )
Mouse FLAG (Sigma Cat # F3165-.2MG, Specificity: recombinant protein; for WB; PMID: 19615732)
Rabbit UBXD7 (Thermofisher scientific; Cat# PA5-61972; Specificity: Human; for WB; PMID: 29685906),
Rabbit Histone-H3 (Abcam; Cat# ab1791; Specificity: Mouse, Rat, Human, Saccharomyces cerevisiae, Xenopus laevis, Arabidopsis thaliana, Drosophila melanogaster, Indian muntjac, Schizosaccharomyces pombe; for WB; PMID: 33270882)
HRP conjugated anti-rabbit (H+L) (Promega; W401B; WB: 1:10,000; PMID: 22389506).
HRP conjugated anti-mouse (H+L) (Promega; W402B; WB: 1:10,000; PMID: 22389506).
Goat anti-Mouse IgG (H+L) Cross-Adsorbed Secondary Antibody, Alexa Fluor™ 568 (ThermoFisher Scientific; Catalog # A-11004; IF: 1:10,000; PMID: 36350286).
Goat anti-Mouse IgG (H+L) Cross-Adsorbed Secondary Antibody, Alexa Fluor™ 488 (ThermoFisher Scientific; Catalog # A-11001; IF: 1:10,000; PMID: 36526630)

# Eukaryotic cell lines

Policy information about cell lines

| | |
|---|---|
| Cell line source(s) | HEK293T cells (ATCC# CRL-3216™) , and COS7 cells (ATCC# CRL-1651™) were procured from ATCC.<br>HeLa Kyoto (CLS Cell Lines Service GmbH; catalog number 300670) is a gift from Ron Kopito, Stanford University.<br>HeLa-Flp-IN-TREX cell line (ThermoFisher Cat# R71407) with introduced Flp-In site (Flp-In™ T-REx™ Core Kit, Cat# K650001; Thermofisher Scientific) is a gift from Brian Raught, University of Toronto.<br>HEK293 (ATCC# CRL-1573™) is a gift from James Olzmann, University of California Berkeley.<br>Mouse embryonic fibroblasts were obtained from Mice carrying p97 R155H mutations from Weihl lab. |
| Authentication | Cell lines were not authenticated. |
| Mycoplasma contamination | All cell lines were negative for mycoplasma based on PCR or Myco-Alert (Lonza) |
| Commonly misidentified lines<br>(See ICLAC register) | No commonly misidentified cell lines were used in the study |

# Animals and other organisms

Policy information about studies involving animals; ARRIVE guidelines recommended for reporting animal research

| | |
|---|---|
| Laboratory animals | C57BL/6 (stock No.: 000664) and p97R155H/WT (B6;129S-Vcptm1Itl/J, Stock No: 021968) were purchased from Jackson Laboratory. p97R155C/WT and p97 cKO (VCPFL/FL; CaMKIIa-Cre) were obtained as reported previously54,55. All mice utilized in the study were on a C57BL/6 background. Both male and female mice were used in this study and age of mice are mentioned in figure legends. All mice were housed in a pathogen-free environment under controlled environmental conditions with 12 h light-dark cycles at humidity 30-70%, temperature 20-26°C (68-79°F) with ventilation sufficient to maintain appropriate temperature and humidity ranges and to control odor, where they received food and water ad libitum until being sacrificed. Animal procedures were performed in accordance with protocols approved by the Institutional Animal Care and Use Committee (Animal Welfare Assurance # D16-00245; Protocol No. 22-0298) at Washington University School of Medicine. |
| Wild animals | no wild animals were used in the study. |
| Field-collected samples | no field collected samples were used in the study |
| Ethics oversight | Animal procedures were performed in accordance with protocols approved by the Institutional Animal Care and Use Committee (Animal Welfare Assurance # D16-00245; Protocol No. 22-0298) at Washington University School of Medicine. |

Note that full information on the approval of the study protocol must also be provided in the manuscript.
